# Evaluation of the efficacy of radiation-modifying compounds using γH2AX as a molecular marker of DNA double-strand breaks

**DOI:** 10.1186/2041-9414-2-3

**Published:** 2011-01-25

**Authors:** Li-Jeen Mah, Christian Orlowski, Katherine Ververis, Raja S Vasireddy, Assam El-Osta, Tom C Karagiannis

**Affiliations:** 1 Epigenomic Medicine, Baker IDI Heart and Diabetes Institute, The Alfred Medical Research and Education Precinct, Melbourne, Victoria, Australia; 2 Department of Pathology, The University of Melbourne, Parkville, Victoria, Australia; 3 Epigenetics in Human Health and Disease, Baker IDI Heart and Diabetes Institute, The Alfred Medical Research and Education Precinct, Melbourne, Victoria, Australia; 4 Department of Anatomy and Cell Biology, The University of Melbourne, Parkville, Victoria, Australia; 5 Department of Medicine, Monash University, Melbourne, Victoria, Australia; 6 Epigenomic Profiling Facility, Baker IDI Heart and Diabetes Institute, The Alfred Medical Research and Education Precinct, Melbourne, Victoria, Australia

## Abstract

Radiation therapy is a widely used therapeutic approach for cancer. To improve the efficacy of radiotherapy there is an intense interest in combining this modality with two broad classes of compounds, radiosensitizers and radioprotectors. These either enhance tumour-killing efficacy or mitigate damage to surrounding non-malignant tissue, respectively. Radiation exposure often results in the formation of DNA double-strand breaks, which are marked by the induction of H2AX phosphorylation to generate γH2AX. In addition to its essential role in DDR signalling and coordination of double-strand break repair, the ability to visualize and quantitate γH2AX foci using immunofluorescence microscopy techniques enables it to be exploited as an indicator of therapeutic efficacy in a range of cell types and tissues. This review will explore the emerging applicability of γH2AX as a marker for monitoring the effectiveness of radiation-modifying compounds.

## Introduction

Radiotherapy is widely used for the management of cancer and relies on ionizing radiation (IR)-induced DNA damage to kill malignant cells. DNA double-strand breaks (DSBs), which are exceptionally lethal lesions can be formed either by direct energy deposition or indirectly through the radiolysis of water molecules, which generate clusters of reactive oxygen species that attack DNA molecules [1-4]. DSBs are essentially two single-stranded nicks in opposing DNA strands that occur in close proximity, severely compromising genomic stability [2,5-7]. Therefore, it is critical that DSBs are repaired quickly and efficiently to prevent cellular death, chromosomal aberrations and mutations [6,8]. A series of complex pathways collectively known as the DNA damage response (DDR) is responsible for the recognition, signalling and repair of DSBs in cells, ultimately resulting in either cell survival or cell death [9,10]. DSBs are repaired by two major pathways, homologous recombination (HR) or non-homologous end joining (NHEJ), each with distinct and overlapping roles in maintaining genomic integrity. NHEJ, the more error-prone pathway, is commonly employed following IR-induced damage [11]. IR-induced DSBs cause rapid phosphorylation of the histone H2A variant H2AX to form γH2AX. This phosphorylation event takes place at the highly conserved SQ motif, which is a common substrate for the phosphatidyl-inosito 3-kinase (PI3K) family of proteins including ataxia telangiectasia mutated (ATM) [12-16]. Discrete nuclear foci that form as a result of H2AX phosphorylation are now widely used as a sensitive and reliable marker of DSBs [17,18]. Following a discussion of the biology of γH2AX formation, this review will focus on the utility of γH2AX as a molecular marker for monitoring the efficacy of radiation-modifying compounds.

### Radiation-induced γH2AX formation

Recent years have witnessed a remarkable proliferation in immunofluorescence-based assays dedicated to the visualization of γH2AX foci. This has emerged as the preferred method of DSB detection given that 20-40 DSBs are estimated to form per Gray of γ-radiation [17,18]. Due to its high sensitivity, DSBs can be distinguished at clinically relevant doses, unlike previous methods which required lysis at high temperatures or large doses of IR (5-50Gy), which are well above biologically relevant doses [19,20]. γH2AX foci detection allows the distinction of the temporal and spatial distribution of DSB formation and can be detected just minutes after γ-radiation, reaching a peak between 30-60 minutes post-irradiation and typically returning to background levels (at relatively lower doses) within 24 hours [2,21]. Comparisons of foci numbers in irradiated or treated samples are made in comparison to appropriate controls and background levels in the cell lines of interest. Interestingly, embryonic stem cells have very high intrinsic levels of γH2AX (more than 100 foci per nucleus) compared to cancer lines (typically 5-20 foci per nucleus) and normal cell lines (typically 1 or 2 foci per nucleus) [22-24]. The characteristics of γH2AX formation have been most widely investigated in the context of γ-radiation-induced DNA double-strand breaks, however, γH2AX has also been evaluated following irradiation with high linear energy-transfer (LET) radiations, such as α-particles and heavy ions [25-30]. The γH2AX staining patterns and kinetics observed with high LET radiation differ significantly compared to those from γ-rays, with the major observations being clusters of foci along the ion track that typically exhibit prolonged repair kinetics [30].

Apart from immunofluorescence, Western blotting has also been suggested as another possible method of evaluating the γH2AX response to IR exposure. Data from our laboratory and others have demonstrated that although Western blotting is able to detect the presence of protein in chromatin extracts, it is disadvantaged by the fact that apoptotic cells express extremely high levels of γH2AX, as assessed by immunofluorescence, making it impossible to distinguish between the γH2AX responses of live cells and apoptotic cells [31]. Furthermore, it is very difficult to accurately detect the discrete differences that are typically observed, particularly when the effects of radiation-modifying compounds are being investigated.

Given its unique sensitivity and specificity, immunofluorescence-based detection of foci formation can potentially act as an accurate biodosimeter for exposure to IR following radiation therapy in cancer patients and is a minimally invasive method that requires only the collection of peripheral blood lymphocytes or skin biopsies [32,33]. Assays of γH2AX foci numbers can serve as an indication of the efficacy of various cancer therapies as well as aid in the observation of individual patient radiosensitivities and responses to specific radiation-modifying agents [34-36]. It is important to note, however, that scoring individual γH2AX foci is most accurate at doses below 4Gy (unless DSB repair, for example, 24 hours after irradiation, is being investigated in which cases much higher doses can be examined). When examining initial damage γH2AX foci typically overlap above 4 Gy, as has been shown in our laboratory and others [37]. Methods involving quantitation of total nuclear fluorescence have been employed for monitoring γH2AX formation at higher doses.

### γH2AX foci form preferentially in euchromatin

One striking observation about γH2AX is that foci are rarely detected at heterochromatic sites, which typically demonstrate resistance to IR-induced γH2AX foci formation in spite of heterochromatin's rich DNA content [38-41]. Chromatin immunoprecipitation (ChIP) analysis revealed that transcriptionally silent heterochromatic regions are resistant to γH2AX accumulation in both mammalian and yeast cells [38,42]. γH2AX foci distribution within irradiated cells is uneven as foci can only be detected at the periphery of heterochromatic regions rather than within them, the boundaries of which are maintained by methylation of lysine at position 9 on histone H3 (H3K9), an important epigenomic imprint of heterochromatic regions [43,44]. The noticeable lack of H2AX phosphorylation within heterochromatic regions may be attributable to lower vulnerability of compacted DNA to DSB induction, migration of DSBs to the periphery, lower amounts of available H2AX, or possible epigenetic mechanisms that operate in the region to restrict the accessibility of kinases responsible for H2AX phosphorylation [40]. Epigenetic mechanisms appear to be the best possible explanation for the refractory nature of heterochromatin to γH2AX generation as histone deacetylase inhibitors (HDACi) have been shown to influence chromatin reorganisation, forcing the movement of DSBs to the periphery of heterochromatic regions [38,45]. Another probability could be that γH2AX foci are epigenetically shielded by loss of heterochromatin features and local chromatin decondensation at DSB sites [46]. With respect to radiomodification, numerous emerging compounds, such as the HDACi discussed below, alter chromatin architecture. Therefore, the use of γH2AX as molecular marker of DSBs in combination with epigenetic markers of euchromatin and heterochromatin would allow correlation of radiomodification and changes in chromatin landscape when investigating relevant compounds.

### Radioprotection

One of the major hurdles with respect to radiotherapy use is the preservation of normal tissue while still ensuring the effective killing of tumour cells. Hence, the radiation dose must be limited by the tolerance of non-tumour cells to minimise toxicity to normal, healthy tissue [47]. The issue of therapeutic efficacy has been an important one to address, bringing about the identification and development of compounds such as radiosensitizers and radioprotectors, which either sensitize tumour cells to IR or protect normal cells, respectively [47]. Combining radiotherapy with these radiation-modifying agents is useful in improving therapeutic gain while reducing unintended collateral damage to surrounding normal tissue. Here we discuss, two classes of commonly investigated radioprotectors, the free radical scavengers including amifostine and tempol and the emerging DNA minor groove binding radioprotectors.

Among the first radioprotectors discovered were the sulfhydryl compounds in the early 1950s [48,49]. Amifostine (WR-2721) is a well-characterised radioprotector approved by the US Food and Drug Administration (USFDA) for the reduction of cisplatin-induced cumulative renal toxicity in ovarian cancer patients and xerostomia in head and neck cancer patients [50,51]. Amifostine is a thiol that confers radioprotection against the toxicity associated with radiation without reducing the efficacy of radiotherapy due to its ability to selectively scavenge radiation-induced radical oxygen species (ROS) before they harm the vulnerable DNA of normal cells [52-54]. Although the extent of radioprotection varies in different tissues, amifostine has broad-spectrum properties that protect non-tumour cells originating from almost all tissue types [50,53-55]. Its selectivity for normal tissue is due to its preferential accumulation in normal tissue compared to the hypoxic environment of tumour tissues with low pH and low alkaline phosphatase, which is required to dephosphorylate and activate amifostine [56,57]. The active metabolite, WR-1065 scavenges free radicals and is oxidised, causing anoxia or the rapid consumption of oxygen in tissues [58]. Amifostine may also cause chromatin compaction, reducing possible sites for ROS activity, thus reducing double strand break (DSB) induction as well as increasing DNA repair and cellular proliferation to aid in the recovery of damaged cells [50]. Maximal radioprotection is observed when amifostine is administered within half an hour before radiation exposure [59,60]. Interestingly, it has been shown that the radioprotective properties of amifostine correlated with a reduction in γH2AX foci in human microvascular endothelial cells [61]. However, this same paper called the use of γH2AX as molecular marker for evaluating the efficacy of radioprotectors into question since the antioxidants N-acetyl-l-cysteine, captopril and mesna protected from radiation-induced γH2AX formation but did not exhibit radioprotective properties by clonogenic survival [61].

Tempol (4-hydroxy-2,2,6,6-tetramethyl-piperidinyloxy) belongs to a class of water-soluble nitroxides which are membrane-permeable stable free radical compounds that confer protection against radiation-induced damage [62-64]. It is thought to elicit its effects through the oxidation of reduced transition metals, scavenging free radicals and mimicking superoxide dismutase activity [63,65]. *In vitro *studies have shown that tempol has dose-dependent radioprotective properties which are more efficacious in aerobic conditions as compared to hypoxic environments [66]. Tempol is capable of protecting cells from the mutagenic effects of oxy radicals, aminoxyls and nitroxyls, decreases X-ray induced DSB frequency, and reduces the number of chromosomal aberrations in human peripheral blood cells [67-69]. These findings were also observed *in vivo *and tempol was shown to be specific for non-tumour cells, which may be attributable to the lack of oxygen or high levels of bioreduction occurring in tumour cells [70]. However, these effects are observed only if tempol is administered immediately before radiation exposure [71]. Interestingly, γH2AX has been employed as a molecular marker to evaluate the effects of tempol in the context of radiation-induced bystander effect [72]. Tempol was found to reduce γH2AX formation in normal human fibroblasts that were exposed to media from UVC-irradiated cells [72].

The DNA minor groove binding bibenzimidazoles represent a different class of potential radioprotectors. Essentially, this group of experimental compounds can be considered as DNA antioxidants that display much greater potency than amifostine and tempol in cell culture systems [73]. DNA minor groove binding radioprotectors are exemplified by the commercially available and widely used DNA stain, Hoechst 33342. Hoechst 33342 binds tightly in the minor groove of DNA predominantly in regions containing four consecutive AATT base pairs [74,75]. The compound is utilised extensively in flow cytometric studies due to its intrinsic fluorescence properties which become amplified once the ligand is bound to DNA. In the early 1980s Hoechst 33342 was shown to possess radioprotective properties which have been subsequently investigated in cell culture systems and *in vivo *[73,76-82]. Synthetic chemistry was employed to improve the radioprotective properties of Hoechst 33342 leading to the development of the potent analogues, proamine and methylproamine [73,77,78]. Cell culture studies using conventional clonogenic survival assays indicate that methylproamine is the most potent of the three analogues [73]. Recently, γH2AX has been used to further evaluate the radioprotective properties of this compound [83,84]. Studies have indicated that methylproamine protects cells from initial DNA damage following ionizing radiation [83,84]. In accordance, it was identified, using γH2AX as a molecular marker of DSBs, that cells must be pretreated with the compound for radioprotection [83,84]. In summary, γH2AX has emerged as particularly useful marker for evaluating the effects of compounds that protect cells from the effects of ionizing radiation and can provide further insights into radioprotective mechanisms.

### Radiation sensitizers

Radiosensitizers enhance the sensitivity of cells to radiation. For example, numerous conventional chemotherapeutics, such as bleomycin, etoposide and the anthracyclines are known to sensitize cells to the effects of ionizing radiation. Doxorubicin is a frontline anti-cancer chemotherapeutic anthracycline which elicits its cytotoxicity through the inhibition of DNA synthesis and DNA topoisomerase II enzymes, chromatin modulation and generation of highly reactive free radicals [85-88]. Tumour resistance and toxicity to normal tissues, especially cardiotoxicity, are major issues in relation to the use of this compound [85]. When combined with radiation, doxorubicin enhances radiosensitivity, especially when administered 4 hours before irradiation [89]. Further evidence of this synergistic effect is highlighted in a clinical study where the combination of radiation and doxorubicin increased response rates and longer remission periods in patients with squamous cell carcinoma of the esophagus, thus increasing patient survival rates [90]. With respect to induction of DSBs and γH2AX, the cytotoxicity of doxorubicin has been widely investigated using this molecular marker. Indeed, one particular study indicated that γH2AX may be used as surrogate marker for clonogenic death induced by doxorubicin and other DSB-inducing genotoxic agents [91]. A recent study has employed γH2AX foci formation to evaluate the DSB-inducing effects of doxorubicin in normal cell cardiomyocytes when used alone and in combination with the HDAC inhibitor Trichostatin A [92]. With respect to combinations of doxorubicin and radiotherapy, an interesting recent finding suggests that low doses of ionizing radiation may suppress doxorubicin-induced senescence as indicated by inhibiting phosphorylation of p38 MAP kinase and p53 [93]. The findings indicated that γH2AX levels remained unchanged prompting the authors to conclude that suppression of doxorubicin-induced senescence was not associated with genotoxic damage [93]. In this study, cells were exposed to doxorubicin four hours after low dose (up to 0.2 Gy) ionizing radiation [93]. Overall, these findings highlight the utility of γH2AX as molecular marker for delineating the combinatorial effects of genotoxic agents and ionizing radiation.

Apart from the classical chemotherapeutics, emerging more selective anti-cancer therapeutics are displaying synergistic, or at least additive effects with ionizing radiation. For example, inhibitors of the DNA damage repair enzyme, poly(ADP-ribose) polymerase (PARP) have been shown to suppress resistance to chemotherapy and to enhance the cytotoxic effects of ionizing radiation [94,95]. PARP inhibitors are particularly effective in targeting cancer cells with mutations in the BRCA1 and BRCA2 tumour suppressor genes [96,97]. Therefore, a number of PARP-inhibiting analogues are currently undergoing clinical trials for BRCA1 and BRCA2 negative advanced breast and ovarian cancers as well as BRCA2 negative prostate cancer [98,99]. BRCA1 and BRCA2 are both implicated in maintaining genomic integrity, at least in part, by their involvement in DNA repair providing a rationale for the effectiveness of PARP inhibitors in malignancies with mutations in these genes [100,101]. Given that PARP inhibitors alter DNA repair, γH2AX has been used as a biomarker for evaluation of the efficacy of these compounds, particularly in combination with other therapeutics, in cancer cell lines [102,103]. Further, γH2AX foci formation has been used to evaluate the combined effects of PARP inhibitors and radiation [94,104]. An exciting new direction is the potential of utilizing poly(ADP-ribosylation) and γH2AX as biomarkers to monitor the effects of PARP inhibitors and combination therapies in clinical samples. This prospect is analysed thoroughly in a recent review [105].

Another, emerging class of potential radiation-modifying compounds that will be discussed is the HDACi. The use of HDAC inhibitors combined with radiation dates back to the 1980s when sodium butyrate was found to potentiate radiosensitivity in cultured cells *in vitro *[106-108]. Several HDAC inhibitors have since proceeded to clinical trials and the USFDA recently approved the use of suberoylanilide hydroxamic acid (SAHA or Vorinostat) for the treatment of cutaneous T-cell lymphoma (CTCL) [109,110]. The molecular mechanisms of action of HDAC inhibitors in enhancing radiation-induced cytotoxicity is thought to involve the transcriptional regulation of genes and impairment of DNA repair processes through the accumulation of acetyl groups on histone and non-histone substrates [109,111-117]. The repression of DDR proteins including ATM, DNA protein kinase catalytic subunit (DNA-PKcs), Rad52, Rad51, p53-binding protein 1 (53BP1) and the tumour suppressor breast cancer 1 (BRCA1) is thought to contribute to cell-killing capacity of HDAC inhibitors [117-121]. Additionally, chromatin remodelling due to HDAC inhibitor-mediated hyperacetylation may inhibit the function of histone deacetylases (HDACs) in the late stages of DNA repair when chromatin is restored to its original state [122]. Another effect of HDAC inhibitor-mediated chromatin remodelling is the generation of a less compacted, relatively open chromatin structure which is more vulnerable to radiation damage [123].

**Figure 1:**
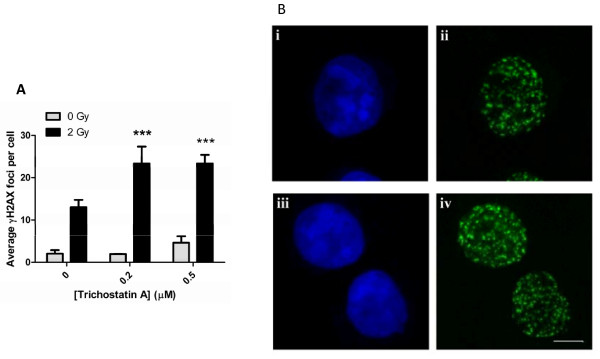
**Trichostatin A enhances radiation-induced γH2AX foci formation in K562 cells**. Cells were pre-treated with the indicated concentrations of Trichostatin A for 24 hours prior to irradiation (2 Gy, ^137^Cs). Cells were fixed and stained for γH2AX analysis 1 hour after irradiation. Images were acquired with a Zeiss LSM 510 meta confocal microscope using 0.5 μm z‐sectioning (63x oil immersion objective). The number of γH2AX foci per nucleus was quantitated using ImageJ (Fiji). Mean ± standard deviations are indicated, *** p < 0.001 (A). Images were exported as TIFF files using Metamorph software for immunofluorescence visualization of nuclei (TO-PRO-3, blue) and γH2AX foci (green). For comparison cells treated with 2 Gy alone (i and ii) and cells exposed to 0.5 μM Trichostatin A prior to 2 Gy irradiation (iii and iv) are shown (B).

Numerous studies involving radiosensitizers such as HDAC inhibitors have used γH2AX as a marker of radiosensitization [121,124-126]. One study investigating the radiosensitizing effects of Trichostatin A (TSA) found that erythroleukemic cell survival was reduced by over 60% when TSA was administered 24 hours prior to γ-radiation exposure, indicating its efficacy in sensitizing cells to radiation. This coincided with a significant increase in preferential euchromatic formation of γH2AX [38,123]. Other studies support this finding, reporting similar observations in glioblastoma cell lines and non-small cell lung cancer cell lines, with a dose-dependent reduction in cell survival and enhanced γH2AX expression [127,128]. Similar findings were observed with other HDAC inhibitors including SAHA, valproic acid and butyric acid [121,126,129-132]. Notably, tumour cells are more susceptible to the cytotoxic effects of HDAC inhibitors compared to normal cells, an important feature of an efficient radiosensitizer [133]. The radiation sensitizing properties of TSA as assessed by γH2AX immunofluorescence are highlighted in Figure 1. These findings are an extension of our previously published chromatin immunoprecipitation studies which highlight the radiation-sensitizing effects the histone deacetylase inhibitor in K562 cells [134].

Paradoxically, HDAC inhibitors have also been shown to possess radioprotective properties. Treatment of cells *in vitro *with phenylbutyrate showed higher clonogenic survival of normal cells which correlated with lower γH2AX foci numbers after radiation exposure, indicating that HDAC inhibitors may reduce radiation damage in normal cells [125]. Phenylbutyrate conferred protection of non-tumour cells against chemically induced oral carcinogenesis and oral mucositis, both severe unwanted side effects of radiation [125].

A well-known issue in radiation oncology is the relative radioresistance of hypoxic cells that exist within solid tumors compared to normoxic malignant cells. Attempts to circumvent the problem associated with tissue hypoxia in radiotherapy include the evaluation of radiation sensitizers, particularly nitroimidazoles, a practise which dates back several decades [135,136]. Numeorus compounds have been identified and evaluated as potential radiosensitisers of hypoxic cells including convetional anticancer chemotherapeutics, bioreductive agents and inhibitors of hypoxia-inducible factor-1 (HIF-1) as reviewed recently [137]. Evaluation of DNA damage using γH2AX as a molecular marker has been employed both in cell culture and *in vivo *studies, to investigate the efficacy of compounds including PX-478 (an HIF-1α inhibitor), nitric oxide (thought to react with free radicals on the DNA), etoposide (classical topoisomerase II inhibitor) and tirapazamine (an hypoxic cell targeting bioreductive cytotoxin) [138-142]. Apart from being a useful marker for the evaluation of the efficacy of radiosensitizers of hypoxic cells, it is noteworthy that a seminal study has identified the critical role of γH2AX and therefore, by extrapolation of the DNA damage response, in hypoxia-induced neovascularization in endothelial cells [143].

### *In vivo *γH2AX models

Overall the *in vitro *studies with radiation protective and radiosensitizing compounds to date highlight the utility of quantitating γH2AX foci as means of examining the efficacy of radiation-modulating compounds *in vitro *as it produces results that, more often than not reflect, data from clonogenic cell survival assays. However, *in vivo *studies to determine the efficacies of radiation-modifying compounds are critical before advancing to preclinical and clinical trials. Radiation therapy results in various tissue-specific effects which can be monitored *in vivo *through a variety of radiobiological models. Among the most well-characterised models are erythema, edema and moist desquamation when the epidermis is exposed to sub-lethal doses of radiation [144,145]. Maximal levels of moist desquamation occur at 20 days post-irradiation, while erythema and edema peak a day or two following radiation exposure [145]. Radiation injury can also be detected using murine colonic mucosal studies as the radiosensitivity of colonic mucosal cells reflects the radiosensitivity of other cells of epithelial origin [146]. Given that the colonic mucosa possesses regeneration capacity, its recovery from radiation injury is a good indicator of the effects of radiation *in vivo *[146]. Another well-established mouse tongue model has been used for studying radiobiological studies on oral mucositis since the early 1990s [147,148]. Oral mucositis is an adverse complication associated with radiotherapy of head and neck cancers. The mouse tongue model allows the evaluation of prophylactic and therapeutic approaches to treatment of oral mucositis [59]. In this model, radiation-induced changes of the mouse tongue epithelium are scored on a daily basis from the onset of first symptoms such as erosions and ulcerations until complete repopulation of the epithelium [148,149].

The models outlined above are well-established and suitable for the monitoring of tissue responses over specified durations however, a major limitation is the need to monitor tissue responses over protracted time periods. Evaluation of γH2AX *in vivo *is emerging as a promising alternative with many studies demonstrating its exquisite sensitivity and reliability [150-152]. Several studies have deduced that γH2AX is a useful indicator for investigating the response of normal and tumour tissues to irradiation as well as for the prediction of individual responses to radiation therapy [150-152]. The immunofluorescence assay has been applied to evaluate DNA damage following irradiation in a range of cell types and tissues, including peripheral blood lymphocytes, skin biopsies and thymic tissues [153-155]. In an interesting recent study, a radiation dose-dependent increase in γH2AX foci was observed in exfoliated buccal mucosal cells following radiation [150]. Given the significance of buccal mucosal cell injury in radiation-induced xerostomia, this model may be suitable for adaptation for the evaluation of potential radiation modifying compounds. Similarly, a recent model indicates the predictive nature of quantitating γH2AX in murine skin following radiation [151]. It was identified that residual foci, 10 days after irradiation, may be the most accurate for determining radiosensitivity [151]. Again, given the clinical problems associated with radiation-induced skin injury, adaptation of this model may provide a means for evaluating the effects of radiomodifiers. However, largely to difficulties in establishing dose-responses that accurately depict radiosensitivity in different cell types and with issues with quantitating γH2AX foci in various cell types in tissue sections (typically 5-8 μm sections and up to 20 μm), widespread use of *in vivo *models for evaluating the effects of radiation-modifying compounds is still limited.

### High through-put screening

The γH2AX immunofluorescence-based assay is currently the most sensitive and robust method for detecting DSBs, prompting research into the development of automated methods to expedite processing and analysis of γH2AX foci [156]. This field is progressing steadily, with developments including automated specimen preparation and computerised image acquisition, digital analysis and computer-based algorithms [153,157,158]. Recently, an automated 96-well immunohistochemistry and microscopy system was unveiled, which can increase the efficiency of γH2AX analysis with reproducible results that correspond to those obtained manually, and could potentially be adapted for high-throughput applications [159].

Large-scale radiological events and the development of new radiopharmaceuticals that modulate radiation sensitivity call for high throughput biodosimetry, utilising γH2AX as a biomarker of DNA damage. Several groups have addressed the need for high throughput evaluation of γH2AX, and one notable advance in this field is the design of an automated system known as RABIT (Rapid Automated Biodosimetry Tool), based on the well-established γH2AX immunofluorescence assay [160]. Peripheral blood mononuclear cells are easily obtained with minimal invasion, have very low levels of background γH2AX expression and low inter-individual variations, validating its use as a tissue sample for damage detection following radiation exposure [19,161,162]. Optimisations are still under way with the development of the RABIT system and its completion will provide a significant boost for the assessment of radiation exposure in humans as well as for monitoring the efficacy of existing and potential radiation-modifying compounds.

## Conclusions

In summary, γH2AX is a widely used molecular marker for monitoring the efficacy of radiation-modifying compounds *in vitro*. However, the assay has not yet surpassed the traditional radiobiological models for preclinical studies with radiation-modifying compounds. On the basis of its popularity in the detection of radiation-induced DNA damage in cell culture studies, and given its reproducibility and reliability, the immunofluorescence assay is likely to become more widely employed *in vivo*. It is expected that with advances in 3D imaging and analysis, superior predictive models of tissue damage based on γH2AX will be established. Finally, it would be a major accomplishment if the assay can be adapted for high-throughput evaluation.

## Competing interests

The authors declare that they have no competing interests.

## Authors' contributions

TCK and AE provided the outline and drafted the manuscript. KV and RSV drafted the radiation-induced and γH2AX formation and γH2AX foci form preferentially in euchromatin sections, respectively and provided the figures for the manuscript. LM and CO drafted the radioprotector and radiation sensitizer sections, respectively. All authors read and approved the final manuscript.
